# From the Gobi to the Sahel: Can China’s anti-desertification model work in Africa?

**DOI:** 10.1007/s13280-026-02363-5

**Published:** 2026-03-19

**Authors:** Annah Lake Zhu, Jesse Rodenbiker, Xiaona Guo, Amadou Ndiaye, Yongdong Wang, Yuan You, Zinabu Bora, Xiaosong Li, Jiaqiang Lei, Ruishan Chen

**Affiliations:** 1 Environmental Policy Group, Wageningen University, Hollandseweg 1, 6706 KN Wageningen, The Netherlands; 2 Department of Geography, Rutgers University-New Brunswick, Lucy Stone Hall, 54 Joyce Kilmer Avenue, Piscataway, NJ USA; 3 School of Design, Shanghai Jiao Tong University, Shanghai, 200240 China; 4 School of Agriculture and Food Sciences, University Amadou Mahtar Mbow, PRM2+QP2, Rufisque, Senegal; 5 State Key Laboratory of Desert and Oasis Ecology, Xinjiang Institute of Ecology and Geography, Chinese Academy of Sciences, Urumqi, 830011 China; 6 International Research Center of Big Data for Sustainable Development Goals, Beijing, 100094 China; 7 Aerospace Information Research Institute, Chinese Academy of Sciences, Beijing, 100094 China; 8 State Key Laboratory of Ecological Safety and Sustainable Development in Arid Lands, Xinjiang Institute of Ecology and Geography, Chinese Academy of Sciences, Urumqi, 830011 China; 9 University of Chinese Academy of Sciences, Beijing, 100049 China

**Keywords:** Africa, China, Desertification, Eco-developmentalism, Ecological restoration, Environmental governance

## Abstract

China’s assistance with Africa’s Great Green Wall offers a critical test case for its eco-developmental approach to combating desertification. Unlike Western-backed conservation models, which focus primarily on ecological restoration, China’s strategy—refined through its Three North Shelterbelt Program—focuses on eco-developmentalism, integrating tree planting with large-scale infrastructure, renewable energy, and livelihood transformation through a broad developmental vision. Comparing longstanding efforts in Senegal with more recent Chinese-backed partnerships in Mauritania, Ethiopia, and Nigeria, we assess the transferability of China’s approach. While technologies like solar-powered irrigation and sand-fixation show promise, their implementation in Africa faces logistical and governance challenges. More fundamentally, the initiative exposes a philosophical divide: Africa’s restoration-focused ambitions versus China’s eco-developmental infrastructure-led model. This tension reflects broader debates about whether and how arid lands should be “restored” or “developed.” We reflect on hybrid approaches that bridge these extremes, while highlighting gaps between China’s technocratic solutions and African institutional realities.

## Introduction

Every three years, hundreds of policymakers, scientists, and development practitioners from across Africa, the Middle East, and Central Asia converge on the arid landscapes of China's western frontiers. Their destination is a global summit on desertification control that forms part of China's Belt and Road environmental cooperation framework. Here, against the backdrop of regenerated deserts and experimental forestry stations, delegates witness what Chinese officials and academics term "the China model" of dryland development (You et al. 2020)—a comprehensive approach to desertification control refined through decades of large-scale socioecological experimentation. The model—one of many “China models” emerging around green development (Liu and Bennett 2024)—is now being actively exported to desertified landscapes across the Global South (Bao et al. 2017; Lei et al. 2025; Li et al. 2025).

This triennial gathering and the “China model” that underlines it represent more than technical knowledge sharing. They embody China's strategic embrace of environmental diplomacy as a pillar of its global governance ambitions. Through initiatives spanning high-level summits to grassroots training programs, China is actively advancing its domestic vision of *Ecological Civilization Building* (生态文明建设)—the overarching framework guiding all environmental policy in the country (Weins et al. 2022; Hughes and Wang 2023; Xue et al. 2023; Zhao et al. 2023)—as a model for global adoption. In doing so, China frames its environmental management practices as scalable and transferable solutions for other developing nations (Zhu 2022; Traore et al. 2025; Zhu et al. 2024). Having established itself as the Global South's premier infrastructure financier, the country is now moving on to environmental diplomacy (Harlan and Lu 2022; Harlan et al. 2025). This involves sharing its large-scale socioecological experimentation as a blueprint for international environmental assistance—with anti-desertification initiatives serving as a key proving ground (Li et al. 2020; You et al. 2021).

At the forefront of this campaign is China’s partnership with Africa's Great Green Wall (Xinhua 2021a, 2021b; Liu 2023a, b). Launched in 2007 under the auspices of the African Union, Africa’s Great Green Wall aims to restore 100 million hectares of degraded land across the Sahel-Sahara region by 2030 while creating millions of green jobs (Goffner et al. 2019). Yet, despite substantial Western funding and technical support, progress has been slow—assessments suggest only about 4% of the target area has been restored (UNCCD 2020). China's engagement with Africa’s Great Green Wall, formalized only in 2017, aspires to remedy some of these shortcomings. The partnership draws explicitly on lessons from China’s own monumental Great Green Wall project—more formally known as the Three North Shelterbelt Program—initiated in 1978 and slated to continue until 2050 (Li et al. 2012). Planned to span three quarters of a century and cover more than a third of the country’s territory, China’s Three North Shelterbelt represents the world’s longest-running state-led afforestation effort and offers a wealth of lessons—both successes and failures—for the African initiative (Liu 2023a, b).

China-Africa cooperation on the Great Green Wall initiative offers a critical test case for examining emerging patterns of South-South environmental cooperation. Designated as one of ten priority projects in Sino-Africa cooperation (MFA 2024), this partnership ranges from on-the-ground technical support in sand fixation^1^ and solar-powered irrigation to satellite-based geospatial analysis via SDGSAT-1 (the world’s first satellite dedicated to achieving the UN Sustainable Development Goals). It reveals the multiple levels through which China is now striving to build a *global* Ecological Civilization by sharing its distinctive approach to dryland development.

Crucially, the Sino-African partnership also reveals how China’s vision of building a global Ecological Civilization differs from Western paradigms. Whereas Western donors funding Africa’s Great Green Wall typically emphasize ecological restoration and maintaining natural fidelity, China promotes what scholars have termed an "eco-developmental" approach (Esarey et al. 2020; Rodenbiker 2021, 2023; Harrell 2023). That is, China integrates ecological and development ambitions to operationalize state power through expansive socio-environmental engineering. Nature, in this model, is not restored to a prior state but actively developed as infrastructure for civilizational progress. This model reflects China’s signature “infrastructural thinking” (Oakes 2019), where ecological and developmental systems merge into unified, large-scale interventions (Yeh 2022). Desert control projects simultaneously serve as integrated forests, renewable energy installations, and transportation corridors, blurring conventional boundaries between environmental protection and economic development. Natural, social, and physical infrastructures are simultaneously engineered via integrated, large-scale eco-developmental ambitions.

As China-Africa environmental cooperation deepens, the case elicits broader questions: to what extent can China's eco-developmental model be translated across divergent sociopolitical contexts? What are the potential benefits and risks of this approach compared to conventional restoration paradigms? This perspective examines these questions through the lens of China's involvement in Africa's Great Green Wall. We first analyze the challenges facing the African initiative without Chinese involvement, drawing on existing literature, field research, and satellite analyses from project implementation in Senegal (Zhu et al. 2025). We show how Africa’s Great Green Wall to date has been implemented largely through an approach based on ecological restoration. We then contrast these approaches with China's domestic experience, highlighting how the country’s Three North Shelterbelt initiative employs a state-led, eco-developmental approach. The penultimate section examines specific Chinese partnerships in Africa, assessing which elements of the "China model" have gained traction and which have not. We conclude by reflecting on the benefits and drawbacks of eco-developmentalism as a strategy for South-South cooperation.

## Africa’s great green wall: Restoring fragile ecologies

Unlike China’s Three-North Shelterbelt Program, which has operated as a centralized, infrastructure-linked initiative since 1978, Africa’s Great Green Wall represents a more recent and fundamentally different approach to dryland restoration. Adopted in 2007 under the African Union, the project originally envisioned a 15-km-wide wall of trees stretching across the Sahel to combat the perceived southward expansion of the Sahara Desert. This framing has since shifted significantly in response to both practical realities and evolving ecological understanding. Scientific studies now show that the Sahara is not systematically advancing southward and that parts of the Sahel have actually experienced modest greening over recent decades due to changes in rainfall patterns (Fensholt et al. 2017; Kaptué et al. 2015; Stith et al. 2016). Consequently, the initiative has moved away from the literal "green wall" concept toward what the FAO describes as "a mosaic of sustainable land use practices" (FAO 2023).

Despite this rhetorical evolution, the initiative’s implementation and evaluation metrics remain heavily focused on conventional afforestation outcomes. Turner et al. (2021) note that project success continues to be measured primarily through quantitative indicators like trees planted, hectares restored, and people trained—metrics that perpetuate the very "wall" narrative the initiative claims to have moved beyond. This contradiction between stated goals and actual practice reflects deeper structural challenges that have hindered progress across all participating countries. A 2020 assessment found that only 4 percent of the initiative’s ambitious 100 million hectare restoration target had been achieved, with even the more modest 25 million hectare target set by the Pan-African Agency for the Great Green Wall^2^ reaching just 16 percent completion (UNCCD 2020).

Two interrelated factors explain this slow progress: financial flows and implementation models. Both of these issues reveal fundamental differences between the initiative as approached in Africa and China. The challenge of finance is often mischaracterized as a simple lack of resources when the real issue lies in the dramatic gap between pledged amounts and funds actually reaching ground-level projects (Zhu et al. 2025). From 2011 to 2019, while donors reported disbursing $870 million for Great Green Wall-related activities, recipient countries documented receiving only $149 million—a staggering 83 percent loss in the financial pipeline (UNCCD 2020). This suggests systemic inefficiencies in the multilateral funding mechanisms that support the initiative, with resources being absorbed by administrative overhead before reaching implementation sites or being slated for Great Green Wall implementation but ending up elsewhere.

The second challenge concerns the limited ecological and developmental impact of the activities that do get funded. Most African Great Green Wall interventions—including tree planting, agroforestry, and farmer-managed natural regeneration—rely exclusively on rainfed systems with minimal infrastructure support. Water conservation techniques like terracing and *zai* pits (half-moon shaped micro-catchments) are common, but large-scale irrigation or integrated development planning remains conspicuously absent. This stands in sharp contrast to China’s approach, where desertification control projects are systematically linked to transportation corridors, renewable energy installations, and water management infrastructure. The limitations of Africa’s rainfed model are evident in survival rates of planted trees, which frequently fall below 30 percent (Turner et al. 2023) and have been shown to drop below 5 percent for even the most drought-resistant species after two years (Wade et al. 2018).

Project implementation in Senegal encapsulates these broader dynamics. Despite being considered the Great Green Wall’s "leading country" (Benjaminsen et al. 2021) and "sole leader" (Magrin And Mugelé 2020), Senegal’s implementation model suffers from the same disconnect between ecological interventions and regional development planning. Funding from major donors like the World Bank and Global Environment Facility passes through multiple bureaucratic layers—from the Pan-African Agency to Senegal’s national Great Green Wall agency in Dakar, then to regional offices, and finally to local sites in the implementation area—with each transfer reducing the resources available for practical restoration work.

**Fig. 1:**
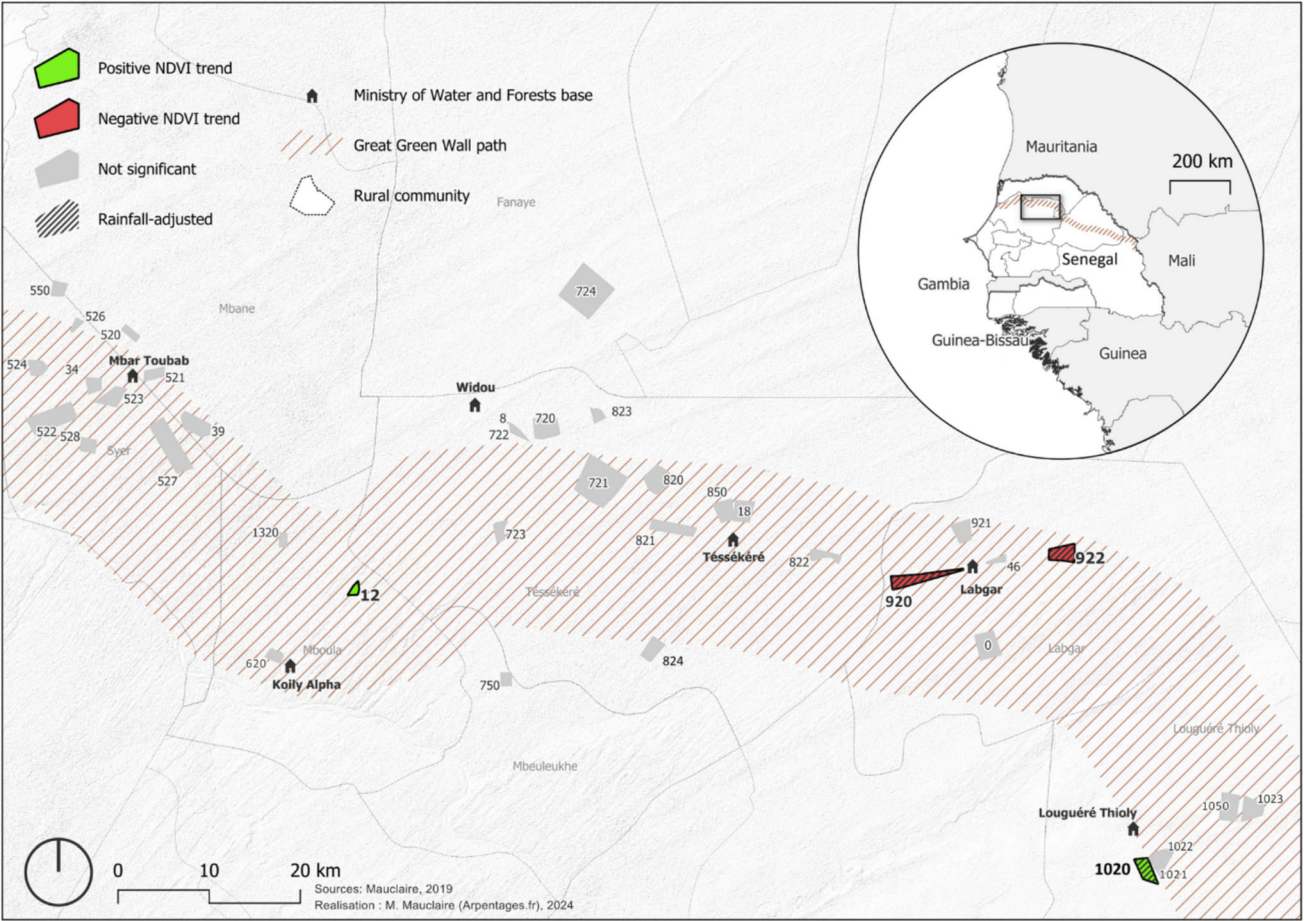
Africa’s Great Green Wall trajectory and reforestation sites in Senegal. Sites with a significant (*p* < 0.05) increasing vegetation (NDVI) trend post-planting are shown in green (site 12). Sites with a significant vegetation trend after being adjusted for what would be expected from rainfall alone are shown in black hatching (red hatched showing decreasing (sites 920 and 922); green hatched showing increasing (site 1020)). Sites with no significant post-planting vegetation trend—the vast majority—are shown in gray. *Source*: Zhu et al. (2025); site location data from Mauclaire (2019)

Impacts on the ground are thus limited, as demonstrated by our team’s satellite analyses of reforestation and restoration sites (Zhu et al. 2025). We tracked vegetation change using satellite imagery for 36 out of 38 intervention sites along the Great Green Wall tract in Senegal from site establishment to the present (see Fig. 1).^3^ Our analysis reveals that only two of these sites showed significant greening trends since planting, with just one exceeding what would be expected from rainfall variability alone (site 1020 in Fig. 1). The vast majority of sites yielded no significant impact on vegetation cover. While Senegal has explored other innovative techniques like farmer-managed natural regeneration, these remain small-scale compared to the country’s primary approach of fencing off reforestation and restoration sites in remote, infrastructure-poor areas.

Ultimately, Africa’s Great Green Wall remains constrained by its conceptual and operational separation of ecological restoration from broader development objectives. Certainly, development has always been assumed as an objective—typically through employment via tree-planting campaigns or higher returns on farming via small-scale water conservation techniques. In some cases, there has been a shift toward even more development-focused initiatives, such as planting fruit and faster growing trees. Yet, in all of these interventions, larger infrastructure developments (irrigation, roads, railways, solar) remain peripheral to non-existent and capital and technology investments are generally quite low. Unlike China’s eco-developmental model—where ecological interventions are integrated within wider infrastructure investments and technological advancement—the African initiative has historically treated these goals as separate rather than synergistic. This fundamental difference, as the following sections will demonstrate, helps explain why China’s approach has gained traction as an alternative framework for dryland development.

## China’s three north shelterbelt: Building eco-developmental infrastructure

While Africa’s Great Green Wall and China’s Three-North Shelterbelt Program have diverged significantly in their implementation, they initially shared similar approaches—albeit at vastly different scales. Formally launched in 1978, China’s initiative builds on revegetation experiments dating back to the 1950s and 60s (Richardson 1990; Bao et al. 2018). Like its African counterpart, China initially focused on mass tree planting as a primary strategy. During the early decades, forestry workers and local residents planted drought-tolerant species at the start of each rainy season, mirroring current practices in Senegal. These efforts, entirely dependent on natural precipitation, faced severe survival challenges, with reported mortality rates exceeding 80% (Chang 2023). The solution—replanting the same areas for three consecutive years—demonstrated both the limitations of rainfed approaches and the extraordinary investment in human capital China was willing to make.

**Fig. 2:**
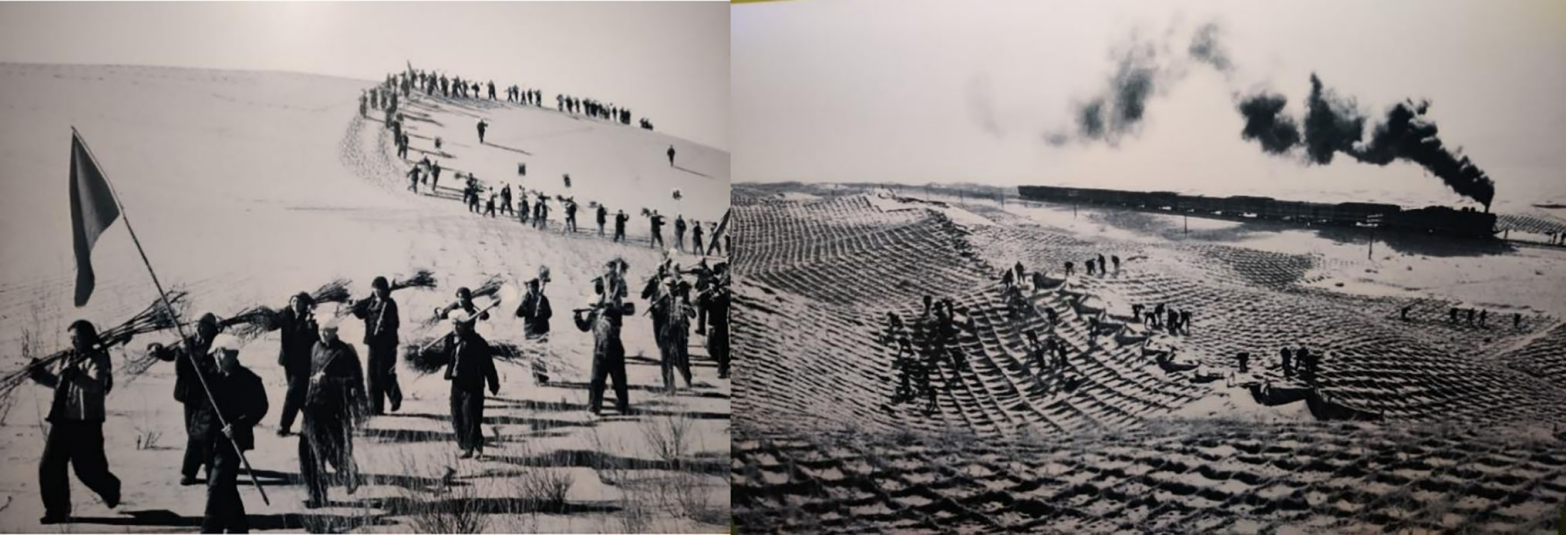
Photos on display at Baijitan Desert Control Museum in Ningxia Province, China, demonstrating straw checkerboarding activities in the 1960s*. * *Source*: Baijitan National Desert Control Museum, 2024

Unlike Africa’s Great Green Wall, China's early anti-desertification efforts extended beyond afforestation to include massive sand stabilization projects (Fig. 2). Straw checkerboard technology (草方格沙障), first conceived in Russia but refined by the Shapotou Desert Research Station in Ningxia in 1957, represented a breakthrough in dune fixation, reducing sand flux by up to 99.5% through labor-intensive installation of 1m × 1m straw grids (Qiu et al. 2004). The technique came to be renowned both domestically (receiving the prestigious National Science and Technology Progress Award—the top prize in China for technology innovation) and internationally (receiving UNEP’s Global 500 Award for Environmental Achievement in 1994 and a “Earth Guardian” Award in 2017). While effective, the technique required hundreds of thousands of workers to implement and maintain, with each installation needing replacement every three to five years. When combined with strategic planting of shrubs within stabilized areas, these interventions could eventually lead to self-sustaining vegetation growth as accumulated organic matter transformed sand into nascent soil.

**Fig. 3:**
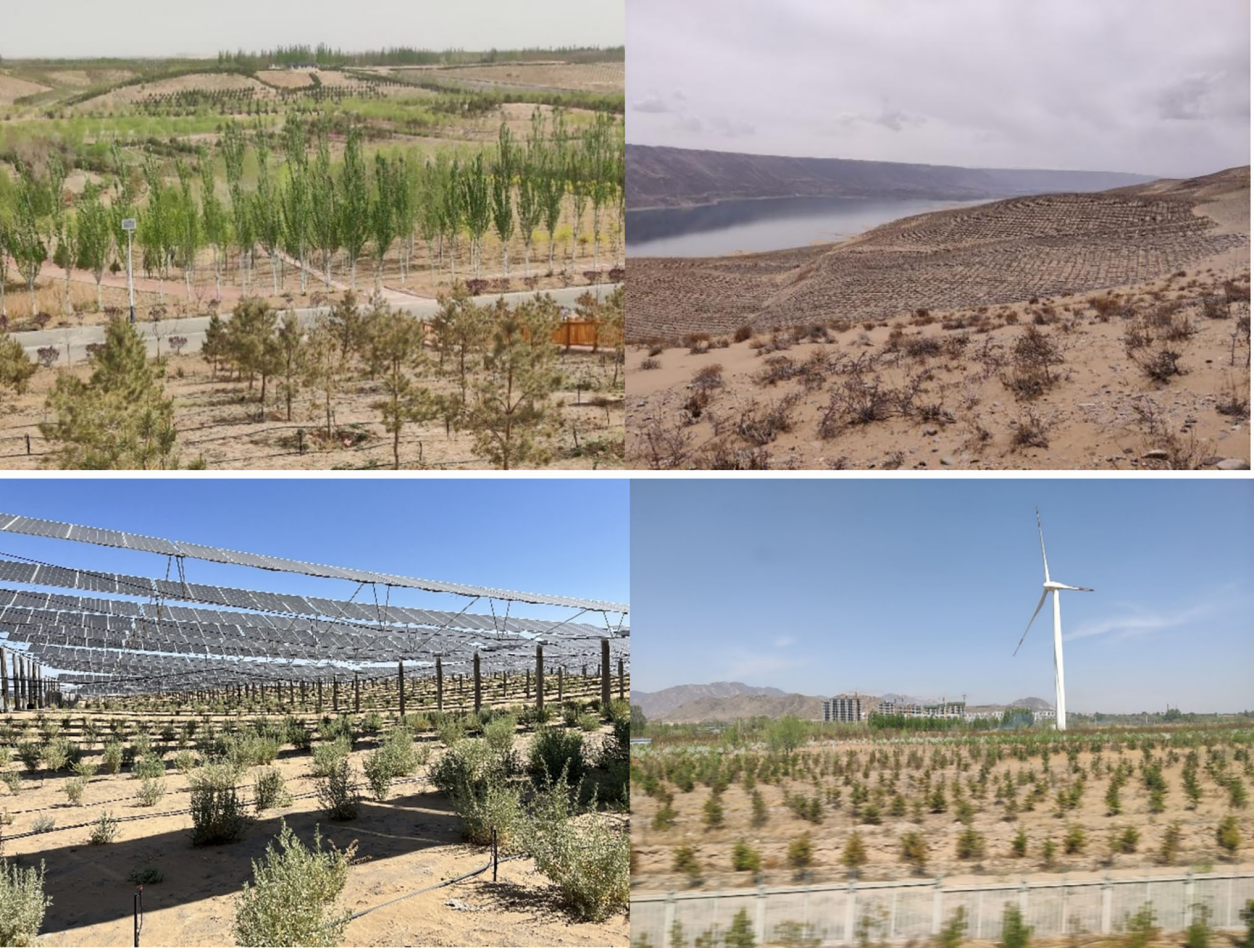
Different planting methods employed through China’s Great Green Wall, including (top left) tree planting by roadsides using drip irrigation in Baijitan, Ningxia Province; (top right) checkerboarding to protect Longyang Gorge Reservoir in Qinghai province; (bottom left) solar-powered drip irrigation in Jiuduntan, Gansu Province; and (bottom right) tree planting integrated with wind power in Zhangjiakou, Hebei Province. Photos by A. Zhu (top and bottom left), R. Chen (bottom right)

Departing from the restoration ethos underpinning Africa’s Great Green Wall, China’s approach to combating desertification—from its earliest iterations—has been fundamentally eco-developmental in character. Rather than restoring natural ecologies to an historically specified pre-disturbance state, China’s afforestation and sand stabilization projects were integrated with large-scale infrastructure development: using trees as shelterbelts for highways (for example, the Tarim Desert Highway), sand stabilization deployed as protective measures for railways (for example, the railway from Baotou to Lanzhou), and so forth (Fig. 3). Unlike Senegal’s isolated reforestation parcels, China’s shelterbelts were designed to safeguard critical assets: newly constructed transportation corridors, water reservoirs, rivers, and the capital city itself.

Roads have been critical to this eco-developmental approach from the beginning, setting it apart from other conventional ecological restoration efforts. This infrastructure-based approach has been encapsulated by the restoration model developed in Chifeng, Inner Mongolia: “using roads to combat desertification, using roads to prosper” (以路治沙、依路致富). In this model, combating desertification is the main objective and roads are first used to that end, breaking up large tracts of land to form a grid-based stabilization network through which people and materials may be mobilized to plant vegetation more effectively. Then, through this newly created system of roads, further development is facilitated via the economies they enable: solar, wind, tourism, drought-resistant agriculture and forestry. This demonstrates how China’s quintessential eco-developmental approach has persisted since its first tree-planting endeavors, though adapted as new technologies have emerged.

As China's economic growth accelerated in the reform era, so too did the scale and technological sophistication of its eco-developmental model. This includes three key technological enhancements. First, mechanization has greatly amplified traditional techniques. The labor-intensive methods of the past have given way to mechanized solutions, such as sand-fixing vehicles capable of installing 40 acres of checkerboards daily, aerial seeding drones that can disperse moisture-retaining seed pods over hundreds of acres, and fully autonomous ground robots that can plant trees ten times faster than humans (Xinhua 2025).

Beyond mechanization, irrigation to support young vegetation growth has helped overcome the rainfed limitations of early efforts. China now boasts a wide array of irrigation strategies from small-scale irrigation to large-scale water-diversion projects. Regions with sufficient groundwater or a nearby water source can employ short-term drip irrigation to support the growth of young drought-resistant tree saplings at their early growth stage before they are strong enough to withstand precipitation variability. At a larger-scale, China also engages in inter-basin water transfers. The South-North Water Diversion Project—the world’s largest inter-basin transfer scheme—is a prime example. The project aims to move 44.8 billion cubic meters of water annually from the Yangtze River Basin to northern and northwestern China by 2050 (Yan et al. 2023). Although this initiative does not support planting efforts directly—indeed, there is explicit guidance against using transferred water to support ecosystem modification—it nonetheless alters the regional water budget. Transferred water is used for municipal and industrial water supply, as well as agricultural irrigation, reducing pressure on local groundwater and river systems in northern China, potentially freeing up water resources for shelterbelts. This luxury is absent in Africa’s rain-dependent model.

Lastly, supplementing mechanization and hydraulic engineering trends, China’s recent integration of renewable energy into desert control represents perhaps the most striking divergence from African approaches (Weselek et al. 2019; Chen et al. 2024a, 2024b). Solar-powered drip irrigation, for example, has been installed to water the vegetative shelterbelt lining the 550-km Tarim Desert Highway, the world’s longest road running through active sand dunes. Although these systems require ongoing maintenance, particularly to prevent sediment from clogging irrigation holes, they represent a remarkable integration of solar power and anti-desertification infrastructure that has yet to be exploited by Africa’s Great Green Wall.

China’s “Solar Great Wall”—a 400-km photovoltaic array currently under construction—provides another prominent example. This installation aims to generate 180 billion kWh annually (equivalent to Beijing’s electricity demand) while reducing sedimentation in the Yellow River (Chen et al. 2024a, 2024b; Hou and Yuan 2024). It also exploits the co-benefits of solar panels acting as a shade for vegetation restoration (Ma et al. 2023; Chen et al. 2024a, 2024b). Recently, the Chinese government issued a Three-North Photovoltaic Desert Control Plan (2025–2030) aiming to install another 0.253 billion KW solar and restore 1.66 million acres of land (Shaw 2025). Solar parks in deserts like Badain Jaran have reportedly increased ground vegetation cover by 74% through microclimate modification and panel runoff utilization (Wang et al. 2024). Where water permits, these installations double as agro-pastoral hubs, hosting medicinal plants, forage crops, and livestock—a far cry from Africa’s fenced, single-purpose tree plots.

This triad of innovations—broad-based mechanization, multiple scales of irrigation, and renewables electrification—reflects the latest iterations within the ongoing evolution of China’s eco-developmental approach to combating desertification. There are, of course, legitimate concerns with such an approach. Ecologically, irrigation and the increasing evapotranspiration associated with vegetating large areas of land may accelerate groundwater depletion and reduce its sustainability (Lu et al. 2018; Zhang and Wu 2020). Over time, China’s large-scale afforestation has been shown even to alter the country’s regional water distribution (An et al. 2025). Socially, such interventions can involve large-scale population movements both voluntarily and sometimes involuntarily, a process labeled “ecological migration” that China has promoted for decades (Zee 2022; Rodenbiker 2023; White 2024). This is enabled by a state-led governance model that engineers social systems as much as ecological (Zhu 2023). In either case, desertification control and the movement of people is not an ecological end in itself, but a platform for developing arid regions. As controversial as these approaches may be, the implications for Sino-African cooperation are profound, suggesting that effective cooperation requires not only understanding current Chinese techniques but the underlying eco-developmental logic that has guided the country’s anti-desertification efforts from the beginning.

## Cooperation between China and Africa

As African nations work toward building the Great Green Wall, China's extensive experience in dryland development provides important lessons—in terms of both the potential and limitations of such socioecological engineering endeavors. Over the past decade, a series of formal partnerships have developed between Chinese institutions and African stakeholders, creating a framework for knowledge exchange and technical cooperation (Li et al. 2025). However, the transfer of China's eco-developmental approach to the African context has proven complex, revealing some promising synergies but also significant challenges rooted in differing socioeconomic and political conditions. Overall, the eco-developmental ethos that pervades the Chinese initiative—alongside the political will and economic investments it requires—has proven far more difficult to translate overseas than the more superficial technological interventions it supports.

**Fig. 4:**
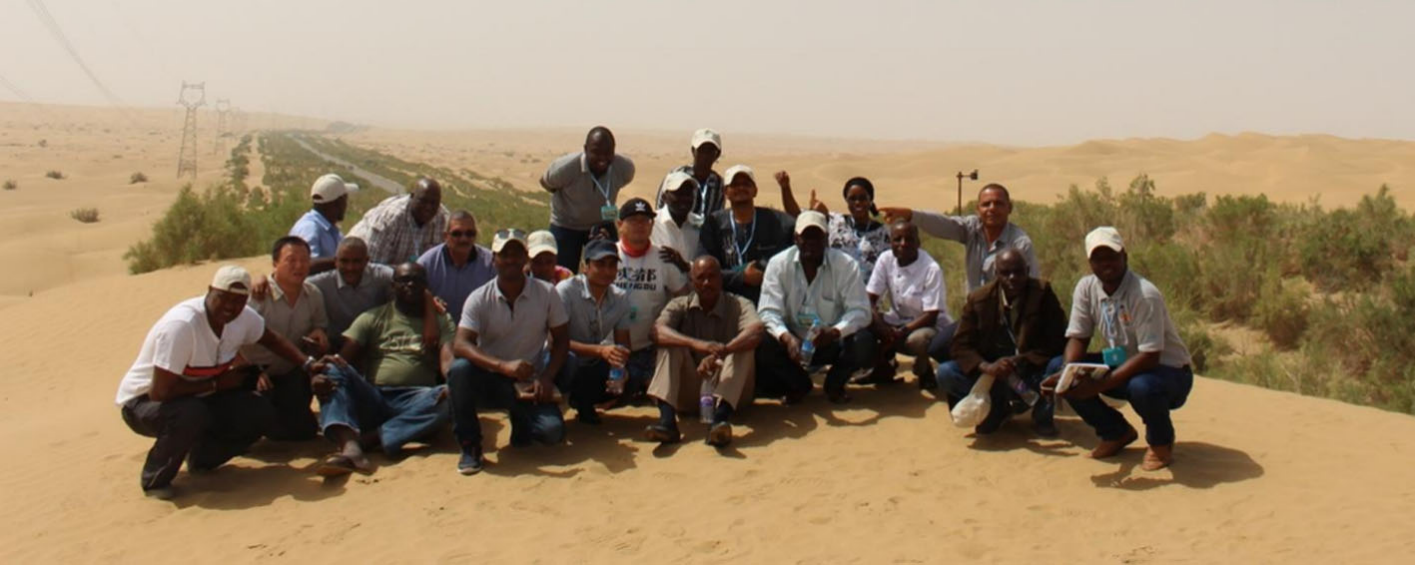
Delegates from Africa’s Great Green Wall countries at the Tarim Desert Highway in Xinjiang, China, 2017. Photo by X. Xu

The recent formalization of China-Africa collaboration on dryland restoration can be traced to several key milestones. The partnership gained official recognition in 2015 when Chinese delegates were invited to participate in the third session of the Pan-African Agency for the Great Green Wall (PAGGW) Summit in N'Djamena, Chad. This marked a turning point as the PAGGW explicitly identified cooperation with China as a strategic priority, acknowledging the potential value of Chinese technologies and methodologies for the African context. Then in 2017, China hosted the UN Convention to Combat Desertification (UNCCD) summit in Ordos, Inner Mongolia—the first time China had hosted such a global environmental conference. The summit served as both a showcase for China's domestic achievements in desertification control and a platform for launching the Belt and Road Cooperation Mechanism to Combat Desertification, inaugurating the institutional framework for ongoing knowledge exchange (Fig. 4), with particular emphasis on adapting Chinese solutions to conditions in developing countries.

A key institutional player in this cooperation has been the Chinese Academy of Sciences’ Xinjiang Institute of Ecology and Geography (XIEG), which signed a landmark agreement with PAGGW in 2017. This memorandum established the framework for collaborative work, including the creation of a West African branch of the Sino-Africa Joint Research Center in Nouakchott, Mauritania—strategically located at the headquarters of PAGGW. The partnership was further strengthened during the 2021 Forum on China-Africa Cooperation, which highlighted the Great Green Wall initiative as a flagship example of South-South cooperation in achieving the UN Sustainable Development Goals.

XIEG's engagement in Africa actually predates these formal agreements, beginning with early collaborations in Libya during 2009–2011 focused on protecting oil extraction and transport infrastructure from sand encroachment. Holding some of the largest oil reserves in Africa, mostly located in remote desert regions, Libya’s roads have experienced the same problems of sand erosion and obstruction endemic to northwestern China. In 2009, the Vice Minister of Agriculture in Libya hosted XIEG staff for a one-month training and by 2011 they had jointly developed designs for shelterbelt projects within the country. Yet, the same year brought the Libyan Civil War and the overthrow of the country’s leader, Muammar Gaddafi, following a NATO-backed intervention. Libya fell into a period of instability that continues to this day. Due to the ongoing political instability and civil unrest, international cooperation was discontinued.

After this setback, XIEG began looking for other partners in Africa, this time eyeing the Sahel. Since around 2011, cooperation began with Mauritania, Nigeria, and Ethiopia—all of which are Sahelian countries that suffer from similar desertification dilemmas and all of which are part of Africa’s Great Green Wall initiative. The 2017 memorandum of understanding with the PAGGW served to enhance this ongoing cooperation, now formally under the banner of Africa’s Great Green Wall. Then in 2019, the cooperation was further enhanced through the UNEP International Ecosystem Monitoring Partnership (IEMP), described on their website as “the first UN Environment Program Collaborative Centre in the South and for the South” (UNEP 2025). XIEG’s cooperation on Africa’s Great Green Wall became part of the UNEP-IEMP project on “Joint research on Practical Technology to Combat Desertification for African Priority Countries of the Great Green Wall,” funded by the Ministry of Science and Technology of China (UNEP-IEMP 2022).

The result has been various, yet still modest, on-site activities across Mauritania, Ethiopia, and Nigeria. Given that Mauritania hosts the PAGGW, partnerships there have been most prominent. This bilateral partnership recently culminated in the latest round of the Taklamakan Desert International Forum held in Nouakchott in December 2025, marking the first time the event was hosted in Africa. The first point of cooperation was the creation of a 3-acre, self-pressurized drip irrigation system next to the agency’s nursery garden intended to preclude the need for extensive manual watering by local residents, leading to better conservation of water and enhancing crop survival rates. Furthermore, if combined with other water management systems, it could eventually utilize rainfall from the wet season for irrigation in the dry season—an innovation that could increase the potential for agriculture in the Sahel (J. Liu 2023a, b).

**Fig. 5:**
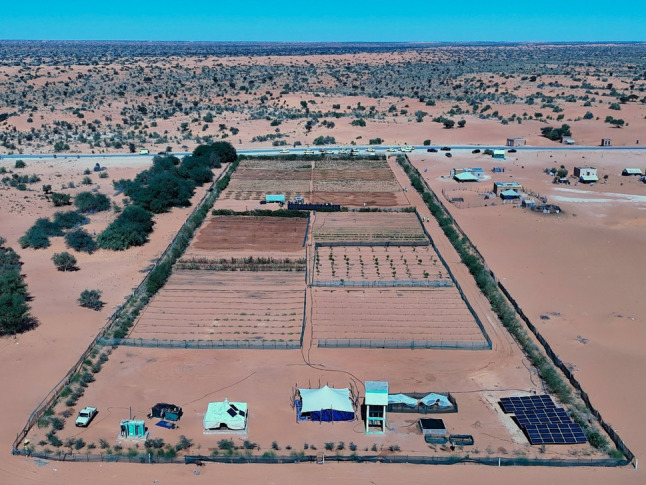
The China-Africa Green Technology Park in the inland Mauritanian city of Bir El Barka, November 2025. Photo by G. Zhu

Since May 2024, the development of a China-Africa Green Technology Park in the inland Mauritanian city of Bir El Barka has also begun (Yuan 2025). The park covers 4 hectares, including solar-powered drip irrigation from groundwater extraction intended to enable year-round agriculture (Fig. 5). It was designed and implemented through cooperation between XIEG, the National Agency for the Great Green Wall in Mauritania, and Sinoway Forest Technology Company—an afforestation enterprise that has long been working in western China and is now expanding its operations overseas to Africa and the Middle East. The prototype is intended as a potential model for cultivating tree saplings and vegetables, supported by China’s Ministry of Science and Technology. In December 2024, at the 16th UNCCD conference, another memorandum was signed to develop 10 000 hectares of carbon-sink forests throughout Mauritania, using the China-Africa Green Technology Park as a prototype (Mauritanian News Agency 2024). This demonstrates future aspirations to scale the model far beyond its current size.

**Fig. 6:**
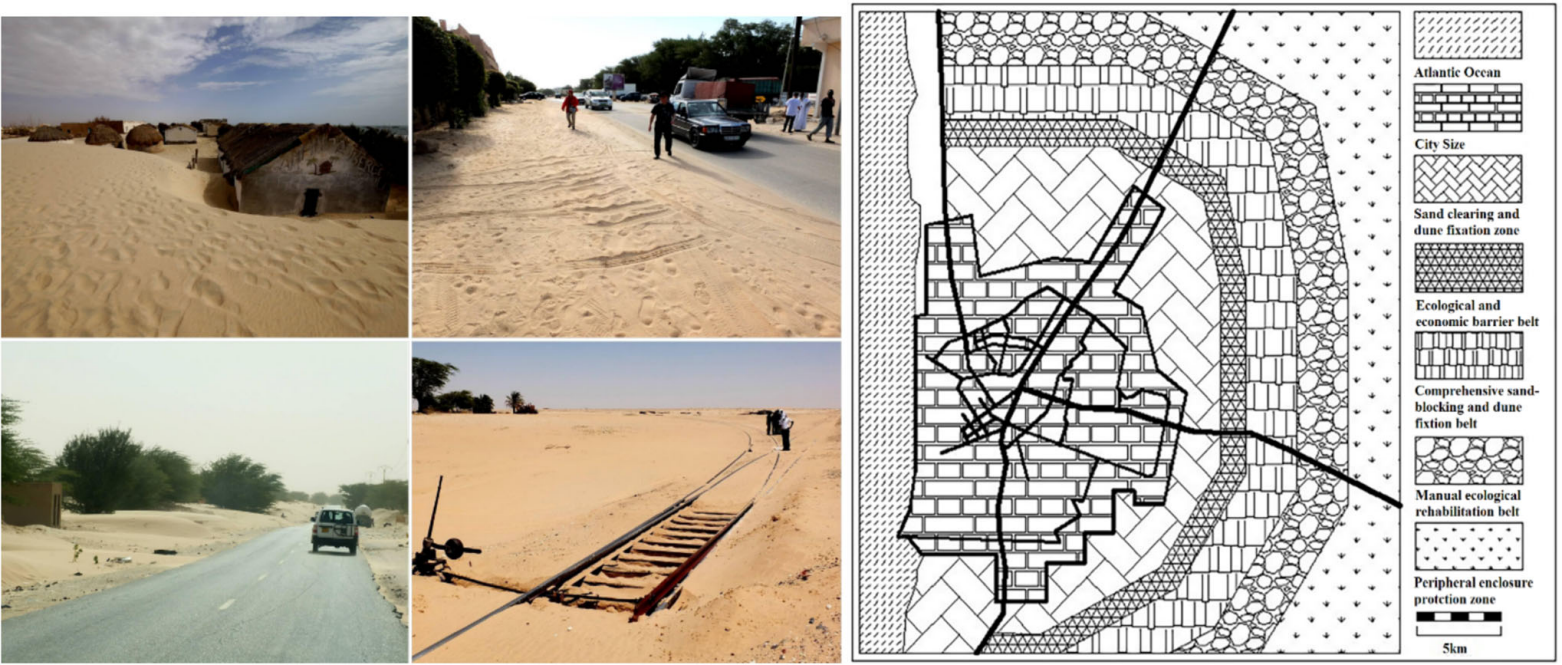
Ecological belts (left) proposed by China’s XIEG to protect Mauritania’s capital city and surrounding areas from sand inundation and infrastructure damage (right). *Source*: XIEG.

Another example of potential eco-developmental cooperation is the "2 districts, 3 belts" sand fixation plan developed for the capital city, Nouakchott. This plan—devised in cooperation with XIEG and based on the famous example in Cele County, Xinjiang—was developed as a comprehensive approach to protecting the capital city from desert encroachment, combining multiple Chinese-derived techniques including a sand-fixation zone surrounding the city. The plan, although not implemented to date, envisions creating concentric zones of protection around the city, starting with immediate sand stabilization measures followed by progressively more complex ecological and economic belts extending over 5 km from the urban boundary (Fig. 6). Implementation would require large-scale ecological transformation of the city's periphery—an endeavor demanding significant political will and administrative capacity that has yet to be mobilized. Nevertheless, native plant species have been identified to cultivate in the nursery for these ecological belts, in addition to ten introduced desert plants from China. These proposed interventions mirror China's eco-developmental approach to urban–rural coordinated planning, which over the last decade has introduced ecological redlines, including large-scale interventions aimed at optimizing specific ecological functions within urban areas (Rodenbiker 2023).

Ethiopia's collaboration with XIEG has taken a different trajectory, focusing on rangeland restoration in the Borana region of southern Ethiopia. Here, bush encroachment has transformed savanna landscapes into dense thickets of woody vegetation with limited pastoral value (Bora et al. 2020, 2021). With XIEG, researchers analyzed historic trends in bush encroachment over the past five decades and established a restoration base to test grasses and clearing methods (Fenetahun et al. 2020; Hare et al. 2020; Yeneayehu et al. 2023). Two demonstration sites were created in cooperation with the Oromia Pastoral Area Development Commission: a 200-hectare restored grassland (doubling forage yield) and a 2-hectare water-management zone using pits and erosion controls. These were later scaled to 50 000 + hectares and 3795 hectares, respectively (UNOSSC 2022). Future work will expand northward into Afar—Ethiopia’s hottest region, where desertification threatens critical infrastructure such as the Ethio-Djibouti Railway and the Addis Ababa-Djibouti Road, which are responsible for shipping most of the country’s imported goods. A 2023 MoU with Samara University and the Ministry of Irrigation and Lowlands Development formalizes plans to study the effects of desertification in the region and potential mitigation measures.

In Nigeria, engagement has maintained a stronger academic focus, centered on Kano State in the country's arid north. A 4-hectare demonstration site showcases four endemic, economically valuable tree species (ebony, locust bean, tamarind, and African black plum), while collaborative research has mapped the spatio-temporal dynamics of land degradation in the region. This work provides important baseline data for future restoration efforts while highlighting the economic potential of native species (Ibrahim et al. 2023; Ogbue et al. 2024).

Beyond physical interventions, China has contributed advanced technological resources through the International Research Center of Big Data for Sustainable Development Goals (CBAS). The center has developed a digital monitoring system for Africa's Great Green Wall initiative, working with African partners and UN agencies. This effort supports both UNEP's SDG monitoring and UNCCD's plans for a global drylands data portal. A key component is China's SDGSAT-1, launched in November 2021 as the first satellite dedicated to SDG monitoring. With its advanced thermal, multispectral, and nighttime imaging capabilities at 10–300 m resolution, the satellite provides crucial data on vegetation changes, urbanization patterns, and land degradation across dryland regions (Guo et al. 2022; Guo 2024).

**Fig. 7:**
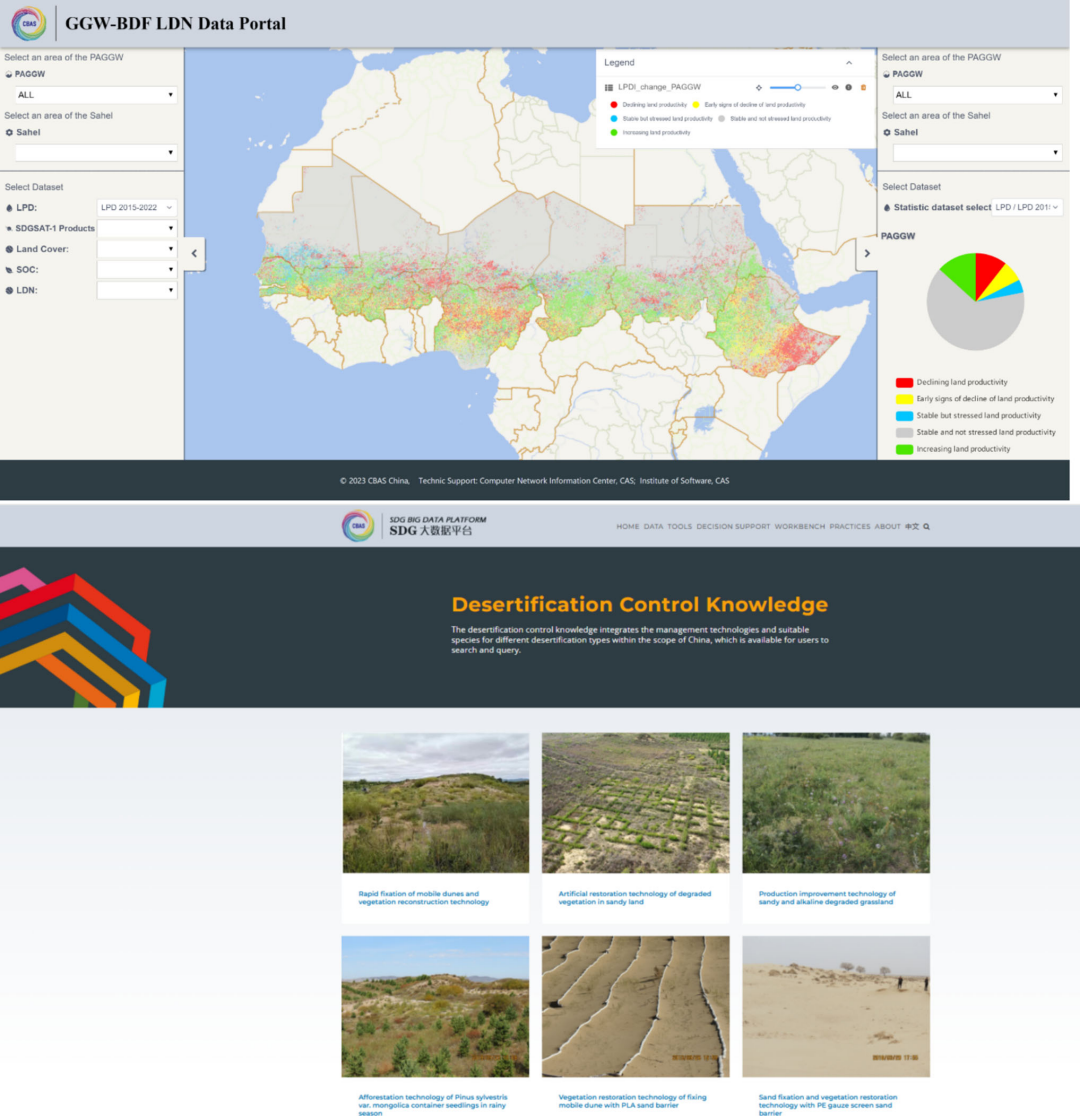
The Great Green Wall Big Data Facilitator, showing (top) land productivity dynamics for Great Green Wall countries from 2015 to 2022, as calculated via the EU-JRC method, and (bottom) a database of Chinese experiences and knowledge production on desertification control. *Source:* https://sdg.casearth.cn/en/onlineTools/GGWBDF

A centerpiece of this cooperation is the *Great Green Wall Big Data Facilitator*, launched in June 2022 (Fig. 7). This open-access tool offers 30-m resolution monitoring—the highest publicly available—for all 11 Great Green Wall countries (Li et al. 2024). While currently only providing silica index data for Mauritania and Nigeria, the platform will eventually incorporate SDGSAT-1 datasets on land cover, soil carbon, and socioeconomic factors. It also includes knowledge-sharing modules featuring China's desertification control techniques (Fig. 7, bottom). To improve accuracy, CBAS is collecting ground-truth data from 2000 sites across five focus countries. Capacity building is also underway, with planned training programs targeting technical staff from Mauritania and Niger, in collaboration with UNCCD, UNEP, FAO and PAGGW secretariat, through what has been called “triangular cooperation” (Zhu et al. 2024). Remote collaboration and satellite investigations are also ongoing at XIEG (Yang et al. 2022).

Overall, these partnerships—whether small-scale pilots or large-scale digital platforms—aim to transmit knowledge and methodologies honed in China over decades. They mark the early stages of a cross-continental collaboration with promise but persistent structural and contextual constraints. Solar-powered irrigation at the China-Africa Green Technology park, for instance, faced challenges ranging from disrupted supply chains for replacement parts to delays linked to religious calendars such as Ramadan. The hope of being able to cultivate tree saplings and agriculture all year round remains unrealized because of water limitations associated with relying on a single borehole. Similarly, the Great Green Wall Big Data Facilitator is an important step toward data-driven governance, yet its relevance depends on systematic ground-truthing, integration with in situ monitoring infrastructure, and sustained user training across African and Chinese institutions. Above all, beyond biophysical differences, the African socio-political and economic context diverges profoundly from that of China, requiring not replication but creative adaptation, experimentation, and institutional imagination.

These divergences are most pronounced in systems of political governance. China’s desertification-control efforts are embedded within a centralized governance imposing long-term policy continuity, mobilizing resources at scale, and enforcing implementation across administrative levels despite social costs (Xue et al. 2023; Zhai et al. 2023; Wu et al. 2024). Since 1978, successive five-year plans, forestry laws, and desertification-control regulations have consistently reaffirmed sand and dust storm mitigation as a state priority. Dedicated funding lines, preferential tax and credit policies, and cadre performance evaluation systems have created a vertically integrated governance chain linking national legislation to provincial and county-level execution. Forestry bureaus, research institutes, and meteorological agencies operate under stable mandates, allowing institutional memory and technical expertise to accumulate over decades.

By contrast, Africa’s Great Green Wall operates within a fragmented governance landscape. Funding remains heavily dependent on short-term external donor cycles, undermining continuity and adaptive learning. Political systems across the eleven Sahelian GGW countries—while diverse—are frequently marked by electoral volatility, military coups, and policy reversals, complicating the execution of long-horizon eco-developmental strategies. Land governance further constrains implementation. Insecure and informal land tenure is widespread in Sahelian GGW zones (Mechiche-Alami et al. 2022), where restoration projects are often implemented on communal lands lacking legally recognized tenure arrangements (Turner et al. 2021). These conditions weaken incentives for long-term stewardship and limit scaling.

China’s experience also highlights the importance of monitoring capacity and bureaucratic feedback loops. Over time, China has constructed a dense monitoring apparatus combining national desertification surveys, forest inventories, extensive meteorological networks, remote-sensing platforms, and digital capabilities (Zhu et al. 2026). These systems enable continuous tracking of vegetation dynamics and policy effectiveness, supporting iterative adjustment. While the Big Data Facilitator for Africa’s Great Green Wall represents a step in this direction, its impact remains limited without integration into routine project monitoring and institutional incentives for data-driven management.

Perhaps most fundamentally, in its recent decades, China’s Three-North Shelterbelt Program unfolded alongside one of the most rapid episodes of economic growth in history. Although the project has always been characterized by its large-scale vision, the economic growth of the past four decades—especially in the 1990s and early 2000s—has made large-scale socioeconomic transformation of the entire country a reality, within which the socioecological transformation of the Three North Shelterbelt constitutes only a small part. This larger socioeconomic context of China’s integration within the global economy has reshaped resource mobilization capacity, land tenure, and technological capabilities, expanding the scope of feasible ecological interventions far beyond what is currently the case in Africa today.

The central question, then, is how can emerging Sino-African cooperation enable selective leapfrogging through context-appropriate approaches rather than wholesale institutional transfer? Current initiatives in the Sahel should be understood as early experiments in cross-pollination, the outcomes of which depend on domestic political will, land governance reforms, and the capacity to integrate ecological restoration into broader development strategies. China’s experience offers not a specific blueprint but a diverse portfolio of lessons—successes, failures, and adaptations—that may inform what is likely to be much smaller-scale experimentation under a plurality of national and sub-national governance conditions in Africa.

## Conclusion: Eco-developmentalism for Africa’s great green wall?

The China–Africa partnership on the Great Green Wall underscores a fundamental divergence in approaches to dryland restoration. While many African-led efforts prioritize ecological recovery through localized interventions—such as isolated planting and regeneration—China advocates for an eco-developmental model that integrates large-scale planting and ecological manipulations within broader processes of infrastructural and economic transformation. This difference is not simply technical but also philosophical. Where Senegal fences off planting zones in remote areas, China develops arid regions by growing vegetation alongside other infrastructure—constructing shelterbelts along highways and massive solar farms amidst vegetation recovery—thus merging ecological and development infrastructure into an integrated eco-developmental vision. This is not to say that such a large-scale, infrastructure-led approach is necessarily better—even less to say that it is necessarily better for the environment—but rather to highlight its growing appeal across the Global South, where development imperatives often outweigh restoration-for-nature’s-sake.

China is beginning to share this eco-developmental approach overseas, as demonstrated most clearly by its cooperation with Mauritania. The partnership envisions solar-powered irrigation and sand fixation around Nouakchott protecting urban development, alongside road and rail corridors leading in and out of the city. While the scale of implementation remains modest compared to China’s domestic transformations, the underlying ethos is clearly expressed: ecological techniques are deployed not as ends in themselves, but as stabilizing means to enable and sustain larger infrastructural investments in harsh environments. Even tools like the Great Green Wall Big Data Platform align with this orientation, privileging technological capacity, productivity, and infrastructural integration over more conventional conservation metrics focused narrowly on ecological outcomes.

For some observers—especially conservation-minded donors in the West—this approach may appear troubling, suggesting industrial-scale transformation of fragile ecologies. The prospect of leveraging ecological elements—tree planting, vegetative shelterbelts, and the like—to ultimately enable large-scale extraction and infrastructure development over the long term, rather than nature restoration for its own sake, challenges dominant conservation paradigms. Yet for many African policymakers who prioritize development over what has been called the “climate blackmail” of strict conservation measures (Monga 2025), the promise of eco-developmentalism resonates more deeply with national aspirations. As leaders like South Africa’s President Cyril Ramaphosa openly frame progress in infrastructural terms—declaring his intent to “turn the nation into a construction site” (S’thembile And Ntando 2025)—China’s approach offers a compelling roadmap.

Realizing this potential, however, requires creative experimentation within country-specific contexts rather than wholesale transfer. Even within China, there is no singular model of eco-development. The road-centered desert restoration strategies of Chifeng noted above, for example, coexist alongside a plethora of other regional models: the “Kekeya model” in Xinjiang, the “Babusha model” in Gansu, the “Baijitan model” in Ningxia, and the “Kubuqi model” in Inner Mongolia, each with their own unique focus and techniques. Despite their differences, all share a common eco-developmental logic that sets them apart from conventional restoration paradigms.

Africa, too, must cultivate its own varied portfolio of approaches. Densely populated nations like Ethiopia and Nigeria, for example, may find greater leverage in rural mobilization and large-scale irrigation, whereas dune-dominated nations, like Mauritania, may benefit more from mechanized sand fixation, shelterbelt highways, and solar-energy integration. Senegal—where industrial agriculture thrives in the irrigated Senegal River Valley while tree planting is relegated to less productive inland areas—could gain from more integrated agroforestry strategies. Meanwhile, communities not oriented toward large-scale infrastructure may benefit most from decentralized, subsistence-focused measures like rainfed gardens and small-scale water harvesting.

At its core, then, the China–Africa collaboration on the Great Green Wall offers an alternative, if not preferable, vision for dryland futures: not the return to a pristine ecological baseline, but the deliberate development of arid landscapes amidst ecological constraints. In this framing, green infrastructure is woven into wider socioeconomic transformations that extend well beyond restoration. At its most generative, this vision does not force a choice between ecological and development thinking. Instead, it demonstrates the potential for socioecological engineering to anchor a new developmental trajectory for Africa’s drylands—one where vegetation grows alongside roads, solar grids, and cities.

## Footnotes

1 Sand fixation is a desertification control technique aimed at stabilizing moving sand dunes and preventing sand drift through biological and mechanical barriers and stabilization systems.

2 The Pan-African Agency for the Great Green Wall is the intergovernmental body that supports implementation of Africa’s Great Green Wall, established under the African Union.

3 Vegetation change was measured using normalized difference vegetation index (NDVI), a widely used remote sensing metric that indicates the amount and health of live green vegetation on a scale of -1 to 1. Two of the 38 sites were excluded from our analysis because they were too small for satellite measurements using the MODIS product.
